# A study on the timing of small-bowel capsule endoscopy and its impact on the detection rate of bleeding sources

**DOI:** 10.1186/s12876-025-04044-1

**Published:** 2025-06-05

**Authors:** Daisuke Kametaka, Masaya Iwamuro, Toshihiro Inokuchi, Seiji Kawano, Sakiko Hiraoka, Motoyuki Otsuka

**Affiliations:** 1 Division of Endoscopy, Okayama University Graduate School of Medicine, Dentistry, and Pharmaceutical Sciences, 2-5-1 Shikata-cho, Kita-ku, Okayama, 700-8558 Japan; 2 Department of Gastroenterology and Hepatology, Okayama University Graduate School of Medicine, Dentistry, and Pharmaceutical Sciences, 2-5-1 Shikata-cho, Kita-ku, Okayama, 700-8558 Japan

**Keywords:** Diagnostic yield, Obscure Gastrointestinal bleeding, Retrospective study, Small-bowel capsule endoscopy, Timing of endoscopy, Vascular lesions

## Abstract

**Background:**

Small-bowel capsule endoscopy (SBCE) is an essential diagnostic tool for obscure gastrointestinal bleeding, particularly for identifying bleeding sources in the small intestine. The timing of SBCE is thought to affect its diagnostic yield; however, the optimal timing remains unknown.

**Methods:**

This retrospective study analyzed 131 patients with overt gastrointestinal bleeding managed with SBCE at our institution between May 2015 and December 2022. Patients were categorized into four groups based on the interval between their last bleeding episode and SBCE: 1–7, 8–14, 15–28, and ≥ 29 days.

**Results:**

Positive findings were observed in approximately 50% of the cases across all intervals, with no statistically significant differences in the detection rates. Vascular lesions were detected primarily within 1–14 days, whereas inflammatory lesions, tumors, and diverticula were identified across all intervals. Notably, 25% of the patients with negative SBCE findings were later diagnosed with sources of non-small bowel bleeding, highlighting the value of follow-up endoscopic evaluations.

**Conclusions:**

Our findings suggest that SBCE can be effective regardless of the time after a bleeding event, contrary to previous recommendations emphasizing its early use. Clinicians should consider performing SBCE whenever feasible to improve the diagnostic outcomes for gastrointestinal bleeding, irrespective of the elapsed time since the last episode.

## Introduction

Gastrointestinal (GI) bleeding is a common clinical challenge with approximately 5% of all cases classified as obscure. Among these, 75% were attributed to small-bowel bleeding [1]. Small-bowel capsule endoscopy (SBCE) has become a cornerstone in the evaluation of suspected small-bowel bleeding owing to its noninvasive nature and ability to provide complete visualization of the small intestine, making it an essential first-line diagnostic tool [2].

Recent studies have emphasized the critical role of timing in maximizing the diagnostic yield of SBCE. Evidence indicates that a shorter interval between the last overt bleeding episode and performance of SBCE is associated with higher diagnostic rates [3–11]. Reflecting these findings, the 2022 European Society of Gastrointestinal Endoscopy (ESGE) guidelines recommend performing SBCE within 48 h of the last bleeding episode, a shift from the earlier recommendation of 14 days [12, 13]. In contrast, the Japanese Guidelines for the Diagnosis and Treatment of Small Bowel Endoscopy advise performing SBCE within 14 days, whereas the American College of Gastroenterology (ACG) guidelines do not specify an interval [14, 15]. These variations highlight the absence of a universally accepted definition of the optimal timing for SBCE.

To address this gap, we examined the relationship between SBCE timing and its diagnostic efficacy. By analyzing data from SBCE performed at our institution, we aimed to identify the optimal interval for detecting bleeding sources. Our findings may contribute to the establishment of standardized guidelines, improve the diagnostic effectiveness of SBCE, and ultimately enhance patient care.

## Methods

### Patients

We retrospectively reviewed the clinical records of 562 patients who underwent SBCE at our institution between May 2015 and December 2022. Patients with overt bleeding were included in this study. The exclusion criteria included failure to swallow the SBCE device, incomplete visualization of the small bowel, and recording errors. Cases involving repeated SBCE in the same patient were included in the analysis. This study was approved by the Ethical Review Board of the Okayama University Hospital (No. 2311-015).

### SBCE examination and assessment of bleeding sources

SBCE was conducted using a PillCam SB3 (Given Imaging Ltd., Yokneam, Israel), and the captured images were analyzed using RAPID Reader 6.5 or 8 software on a RAPID workstation (Given Imaging Ltd.). Except in cases of emergency such as massive bleeding, SBCE was scheduled in the morning following conventional overnight fasting.

The primary endpoint of this study was the detection rate of the bleeding source using SBCE at different time intervals. To conduct this analysis, we categorized the interval between the last bleeding event (hematemesis, hematochezia, or melena) and performing SBCE into four groups: 1–7, 8–14, 15–28, and 29 days or later. Subsequently, the proportion of cases in each interval that yielded positive findings indicative of the bleeding source was calculated. In accordance with the Japanese guidelines, which recommend performing SBCE within 14 days of a bleeding event [14], we initially categorized the patients into four groups based on weekly intervals for analysis. In contrast, as the ESGE guidelines recommend performing SBCE within 48 h [12], we also conducted a subgroup analysis comparing the detection rate of bleeding sources between patients who underwent SBCE within 48 h and those beyond 48 h. Findings were classified using the Saurin classification, in which P1 and P2 lesions were considered positive findings [16].

Patients in whom SBCE failed to identify the bleeding source but had persistent symptoms or strong clinical suspicion underwent additional diagnostic procedures, including upper GI endoscopy, colonoscopy, and contrast-enhanced computed tomography. In cases where SBCE initially yielded negative findings, subsequent investigations revealed bleeding sources outside the small intestine, and the location of the identified bleeding source was reviewed and analyzed.

All statistical analyses were performed using JMP Pro 15 software (SAS Institute Inc., Cary, 144 NC, USA). Categorical variables were tested using the chi-square test. 𝑃 values < 0.05 were considered statistically significant.

## Results

**Fig. 1:**
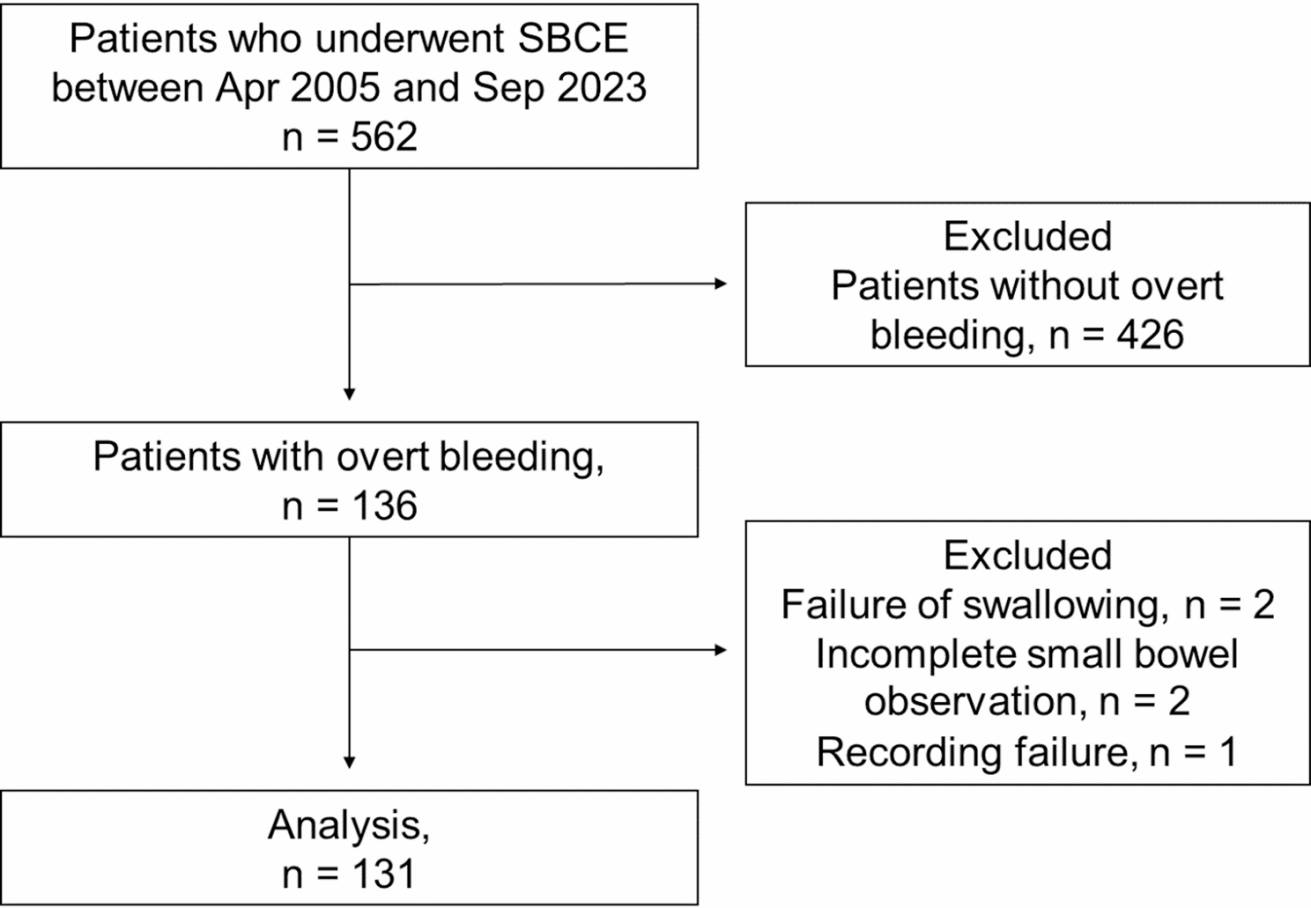
Flowchart of the patients enrolled in this study. Among 562 patients who underwent SBCE using SB3 at our institution between May 2015 and December 2022, 131 patients were enrolled

Among 562 patients who underwent SBCE using the SB3 system at our institution between May 2015 and December 2022, 136 presented with overt bleeding. Five patients were excluded because of failure to swallow the device (*n* = 2), incomplete small bowel visualization (*n* = 2), and recording errors (*n* = 1). Ultimately, 131 patients were included in this study (Fig. 1).

**Fig. 2:**
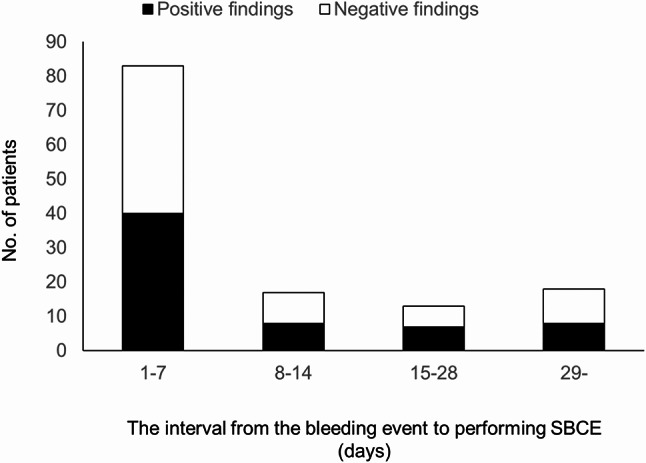
Relationship between the interval from the bleeding event to performing SBCE and rate of positive findings. No significant differences were observed across each interval (*p* = 0.96, Chi-square test)

The median age of the 131 patients (77 men and 54 women) was 69 years (range, 6–89 years) (Table 1). The interval between the bleeding event and performing SBCE was categorized into four groups: 1–7, 8–14, 15–28, and 29 days or later. The most frequent interval observed was 1–7 days (*n* = 83, 63%), followed by 29 days or later (*n* = 18, 14%), 8–14 days (*n* = 17, 13%), and 15–28 days (*n* = 13, 10%). The source of bleeding was identified using SBCE in 63 patients, whereas no source was identified in 68 patients. These were classified as the “positive findings in SBCE” group and the “negative findings in SBCE” group, respectively, for subsequent analyses. The characteristics of the two groups are summarized in Table 1. Notably, the proportion of patients receiving antithrombotic agents was higher in the “positive findings in SBCE” group (26/63, 41%) than in the “negative findings in SBCE” group (16/68, 24%). The rates of positive findings were 48% for 1–7 days (*n* = 40), 47% for 8–14 days (*n* = 8), 54% for 15–28 days (*n* = 7), and 44% for 29 days or later (*n* = 8). These findings indicate no significant differences in the detection rates of bleeding sources among the different intervals from the bleeding event to SBCE (*p* = 0.96, Chi-square test) (Fig. 2). We investigated the 1-year rebleeding rate in the “negative findings in SBCE” group. Among patients who underwent SBCE within 14 days of the bleeding event, the rebleeding rate was 2% (1/52), whereas it was 20% (3/15) in patients who underwent the procedure more than 14 days after the event. The former group had a significantly lower 1-year rebleeding rate (*p* = 0.03) (Table 2). A subgroup analysis comparing the detection rate of bleeding sources between patients who underwent SBCE within 48 h and those who underwent SBCE beyond 48 h also showed no significant difference between the two groups (*p* = 0.39) (Table 3).

**Table 1:** Patient characteristics

	No. of patients	Positive findings in SBCE	Negative findings in SBCE	*P* value
Sex				0.28
Male	77	34	43	
Female	54	29	25	
Median age (years, range)	69 (6–89)			0.49
≥50	107	53	54	
<50	24	10	14	
Timing of SBCE after overt bleeding (days)				0.96
1–7	83	40	43	
8–14	17	8	9	
15–28	13	7	6	
29–	18	8	10	
Liver cirrhosis				0.13
Present	15	10	5	
Absent	116	53	63	
Chronic renal failure				0.85
Present	11	5	6	
Absent	120	58	62	
Cerebrocardiovascular disease				0.15
Present	38	22	16	
Absent	93	41	52	
Anti-thrombotic agent				0.03
Present	42	26	16	
Absent	89	37	52	
Decreased Hb value (g/dl)				0.07
≥ 5	36	22	14	
<5	95	41	54	

SBCE: small bowel-capsule endoscopy, Hb: hemoglobin

**Table 2:** One-year rebleeding rate in the “negative findings in SBCE” group

	Days from bleeding event to SBCE	
	≤ 14	> 14
Rebleeding within 1 year	1	3
No rebleeding within 1 year	51	12

SBCE: small-bowel capsule endoscopy

**Table 3:** Subgroup analysis of the detection rate of bleeding sources based on the interval from the bleeding event to SBCE comparing cases within 48 h and those beyond 48 h

	Interval from the bleeding event to performing SBCE	
	≤ 48 h	> 48 h
Positive findings	22	41
Negative findings	19	49

SBCE: small-bowel capsule endoscopy

**Fig. 3:**
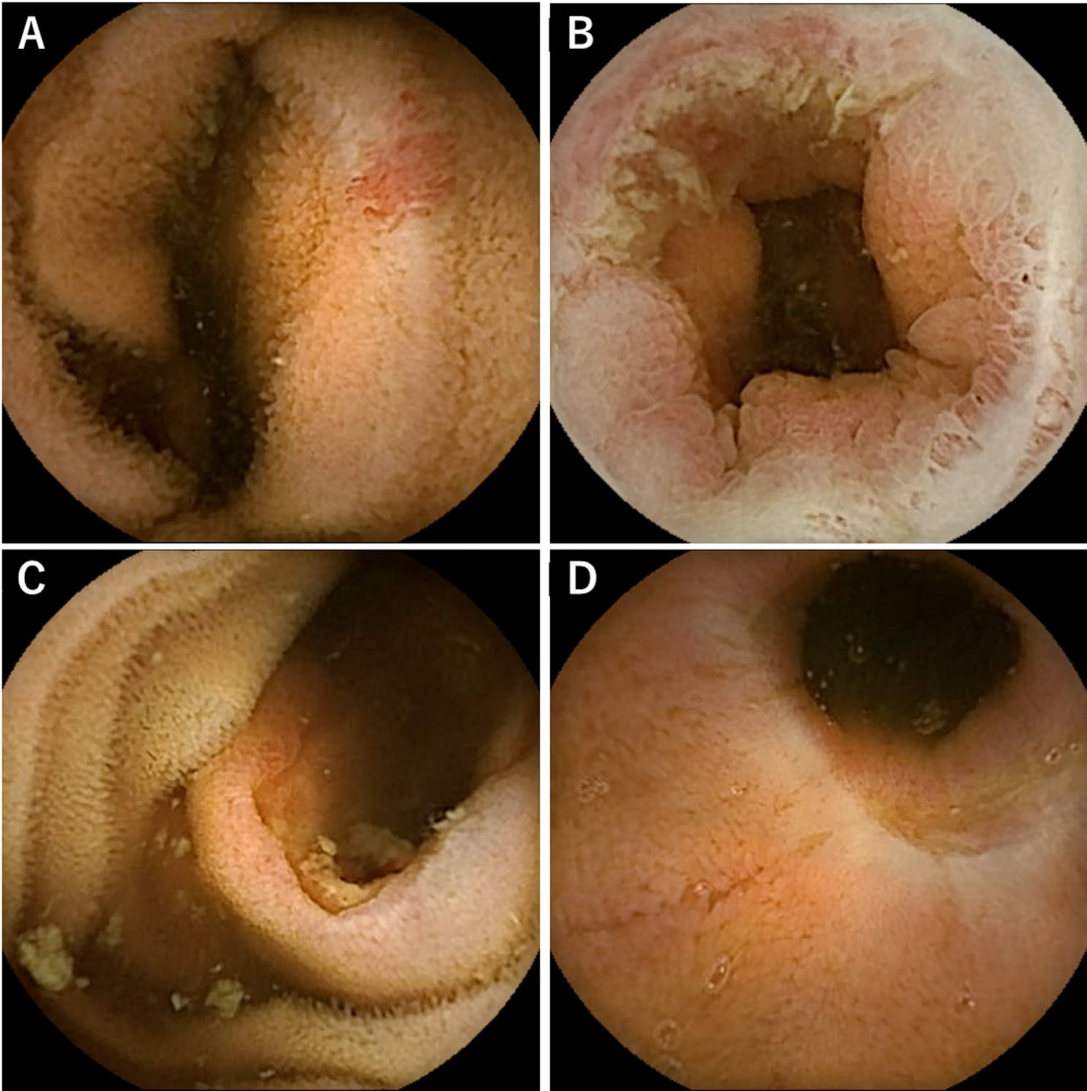
Typical frames of bleeding sources. **A**: angioectasia (vascular lesion). **B**: Crohn’s disease (inflammatory lesion). **C**: Gastrointestinal stromal tumor (tumor). **D**: Meckel’s diverticulum (diverticulum)

**Fig. 4:**
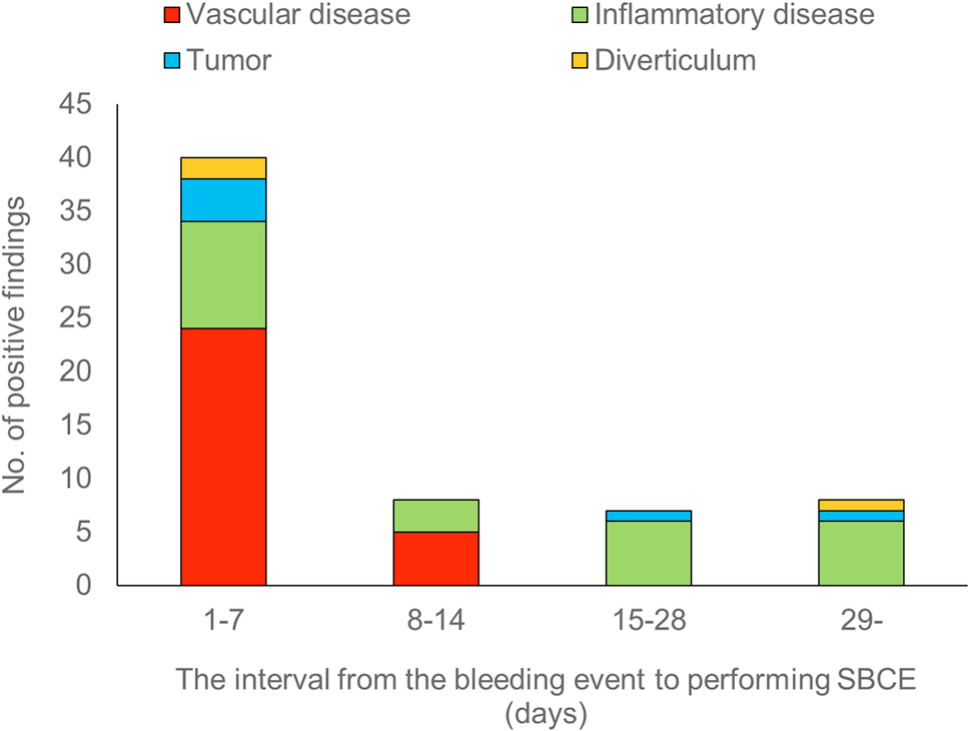
Relationship between the interval from the bleeding event to performing SBCE and types of positive findings. Vascular lesions were identified within 1–14 days, while inflammatory lesions were observed at all time periods. Tumors and diverticula were most common within 1–7 days but remained detectable after 15 days

Positive findings included vascular lesions in 29 (46%), inflammatory lesions in 25 (40%), tumors in 6 (9%), and diverticula in 3 (5%) patients (Fig. 3). All cases of vascular disease were identified in patients who underwent SBCE within 1–14 days. Inflammatory diseases were detected across all the time periods. Tumors and diverticula were most frequently observed within 1–7 days, although they were still detectable after 15 days (Fig. 4). We further examined the distribution of clinical symptoms in relation to the bleeding lesions identified using SBCE (Table 4). Although no statistically significant differences were observed between the clinical symptoms of bleeding lesions, vascular lesions showed a relatively high proportion of cases with a hemoglobin decrease ≥ 5 g/dL (*n* = 15, 52%) and ongoing bleeding at the time of SBCE (*n* = 12, 41%).

**Table 4:** Distribution of clinical symptoms in relation to bleeding lesions identified by small-bowel capsule endoscopy

	Vascular lesions	Inflammatory lesions	Tumors	Diverticula	*P* value
*n*	29	25	6	3	
Hemoglobin decrease ≥ 5 g/dL from baseline, n (%)	15 (52%)	7 (28%)	2 (33%)	1 (33%)	0.34
Syncope at presentation, n (%)	0	1 (4%)	0	0	0.67
Shock status/hemodynamic instability, n (%)	3 (10%)	2 (8%)	0	1 (33%)	0.44
Need for blood transfusion, n (%)	22 (76%)	14 (56%)	5 (83%)	2 (66%)	0.37
Ongoing bleeding, n (%)	12 (41%)	3 (12%)	1 (17%)	1 (33%)	0.10

Among the 68 patients with initially negative findings on SBCE, subsequent investigations identified bleeding sources outside the small bowel in 17 patients (25%). All 17 patients underwent esophagogastroduodenoscopy and colonoscopy prior to SBCE; however, bleeding sources could not be identified during the initial endoscopic examinations. These sources included five cases of angioectasia (three in the stomach and two in the colon), three cases of Dieulafoy ulcers (one in the stomach and two in the colon), three cases of duodenal ulcers, two cases of colonic diverticular bleeding, two cases of gastrojejunal anastomotic ulcers, one case of colorectal ulcer, and one case of gastric polyp (Table 5). The proportion of cases in which the source of bleeding was ultimately identified outside the small bowel was as follows: 26% (11/43) for an interval of 1–7 days from the bleeding event to SBCE, 22% (2/9) for 8–14 days, 50% (3/6) for 15–28 days, and 10% (1/10) for 29 days or more. No statistically significant difference was observed in the proportion of cases with bleeding sources identified outside the small bowel based on the interval between the bleeding event and SBCE (*p* = 0.355).

**Table 5:** Sources of bleeding outside the small bowel identified in 17 patients among 68 with initially negative findings on SBCE

Identified sources of bleeding	Interval from the bleeding event to performing SBCE (days)				
	1–7	8–14	15–28	29–	Total
Angioectasia					
Gastric	2	0	1	0	3
Colorectal	1	0	1	0	2
Dieulafoy ulcer					
Gastric	1	0	0	0	1
Colorectal	1	1	0	0	2
Duodenal ulcer	2	0	0	1	3
Colonic diverticular bleeding	1	1	0	0	2
Gastrojejunal anastomotic ulcer	2	0	0	0	2
Colorectal ulcer	1	0	0	0	1
Gastric polyp	0	0	1	0	1

SBCE: small-bowel capsule endoscopy

## Discussion

In this study, we divided 131 cases of overt bleeding into four intervals based on the time between the last bleeding episode and performance of SBCE and analyzed the bleeding source detection rate for each interval. The positivity rate for identifying potential sources of bleeding was approximately 50% for each interval, indicating no clear causal relationship between the interval and detection rate of bleeding sources. Previous studies suggest that the earlier SBCE is performed for small bowel bleeding, the higher the rate of identifying the bleeding source. According to Bresci et al., the positive detection rate of SBCE for patients suspected of having small bowel bleeding was 91% within 15 days of the last bleeding event and 34% after 15 days [3]. Similarly, Esaki et al. reported a 70% detection rate within seven days, which decreased to 31% after [4]. Based on these findings, the Japanese guidelines recommend performing SBCE within 14 days of the last bleeding [14]. Systematic reviews by Uchida et al. and Estevinho et al. indicated that the diagnostic rate was higher when SBCE was performed within 48 h, that is, within 2 days after the last bleeding [5, 6]. Consequently, ESGE guidelines recommend performing SBCE within 48 h [12]. However, in our study, the positive detection rates were similar across all intervals (approximately 50%). Subgroup analysis comparing the detection rate of bleeding sources between patients undergoing SBCE within 48 h and those undergoing SBCE beyond 48 h also revealed no significant difference between the two groups. Our results showed no significant differences in the positive detection rates among the intervals. Moreover, in cases in which a longer time has passed since overt bleeding, SBCE may still be as useful as when it is performed early. Therefore, SBCE should be performed, regardless of the time elapsed since the last bleeding episode.

In a previous study comparing the 1-year rebleeding rate in patients with negative SBCE findings, those who underwent early SBCE (within 14 days) had a significantly lower rebleeding rate than those who underwent SBCE later (after 14 days) [7]. This finding suggests that early SBCE reduces the risk of missing severe cases. In our study, a similar analysis was conducted, and in the “negative findings in SBCE” group, patients who underwent SBCE within 14 days of the bleeding event had a significantly lower rebleeding rate (*p* = 0.033), which is consistent with the results of prior studies. Therefore, performing SBCE as early as possible, ideally within 14 days of the bleeding event, is recommended to reduce rebleeding.

In our study, > 80% of the patients underwent SBCE within 1–7 days, with a detection rate of 48% for bleeding sources during this period. This detection rate was lower than those reported in previous studies, and several factors may explain this discrepancy. First, although some previous studies have excluded outpatients, this study did not distinguish between inpatients and outpatients [9, 11, 17]. Additionally, a previous study excluded cases later identified as non-small bowel bleeding, whereas this study included such cases in the analysis [4]. Therefore, our study design more closely reflects the actual flow of patients undergoing SBCE for overt GI bleeding, and the data can be considered more representative of real-world clinical practice.

In this study, distinct characteristics were observed for lesions detected within each interval. Vascular lesions were identified only at the early intervals of 1–7 and 8–14 days, whereas inflammatory lesions were observed across all intervals. Tumors and diverticula were detected both in the early interval of 1–7 days, in the later intervals of 15–28 days, and 29 days or later. Similar to our findings, previous reports have indicated that the detection rate of vascular lesions increases with a shorter interval between the last bleeding event and SBCE, whereas reports discussing the optimal detection timing for inflammatory lesions, tumors, or diverticula are not available [3, 7]. In clinical practice, vascular lesions, especially those classified as Types 1a and 2a in the Yano–Yamamoto classification, are often too small to detect in cases of long interval between the last bleeding event and performance of SBCE [18]. By contrast, inflammatory lesions, tumors, and diverticula are less affected by timing, making it difficult to establish a causal relationship between detection timing and these types of lesions. Regarding the timing of SBCE and the characteristics of the identified lesions, another reason for the observed findings is that although no significant differences were observed in clinical symptoms between the bleeding lesions, vascular lesions had relatively higher proportions of cases with a hemoglobin decrease ≥ 5 g/dL (*n* = 15, 52%) and ongoing bleeding at the time of SBCE (*n* = 12, 41%). These results suggest that vascular lesions are associated with more severe bleeding than other types of lesions, which could lead to earlier examination and diagnosis, often within 14 days. Understanding the types of lesions most likely to be detected within each interval is crucial. This knowledge will guide clinicians to focus on specific lesions when reviewing SBCE recordings, thereby enhancing the accuracy and efficiency of the diagnostic process.

Among the 68 patients in whom no bleeding source was identified on SBCE, 17 (25%) were later found to have sources of bleeding outside the small bowel upon reexamination with esophagogastroduodenoscopy or colonoscopy. According to the ACG guidelines, a certain number of cases labeled as potential small bowel bleeding may actually have lesions missed during the initial esophagogastroduodenoscopy or colonoscopy. Second-look endoscopy with esophagogastroduodenoscopy or colonoscopy has been reported to detect lesions in 2–25% and 6–23% of cases, respectively [15]. Furthermore, approximately 30% of cases that were negative on esophagogastroduodenoscopy or colonoscopy who underwent SBCE were later found to have non-small bowel lesions, emphasizing the potential importance of second-look endoscopy before SBCE [19]. In clinical practice, interpreting SBCE images with caution is crucial, considering the possibility of bleeding from sources outside the small bowel, such as the stomach or colorectum, if no clear bleeding source is found in the small bowel. Performing esophagogastroduodenoscopy or colonoscopy again after negative SBCE results should be determined based on the individual case.

This study has several limitations. First, as this was a single-center retrospective observational study with a limited sample size, the findings may have been influenced by selection bias and limited generalizability. The small sample size may have led to a type II (beta) error; therefore, some non-significant results should be interpreted with caution. Additionally, the criteria for performing SBCE and the examinations conducted before and after the procedures varied. However, we believe that our results reflect real-world clinical workflows and provide valuable data regarding the utility of SBCE in clinical practice. A multicenter collaborative study would allow the collection of more extensive data, leading to the accumulation of further evidence and potentially expanding the clinical application of SBCE.

In conclusion, this study found no causal relationship between the interval from the last bleeding event to performing SBCE and positive detection rates. Although SBCE is available only at specialized medical facilities, clinicians should not hesitate to refer patients to these centers regardless of the time elapsed since the bleeding event. Expanding the use of SBCE may improve the identification of bleeding sources and enhance the diagnosis and management of a larger cohort of patients with GI bleeding.

## Data Availability

The datasets generated and analyzed in the current study are available from the corresponding author upon reasonable request.
